# Paediatric Blunt Laryngeal Trauma: A Review

**DOI:** 10.1155/2011/183047

**Published:** 2011-11-13

**Authors:** J. C. Oosthuizen

**Affiliations:** Department of Otorhinolaryngology, St James's Hospital, St James's Gate, Dublin, Ireland

## Abstract

Paediatric blunt laryngeal trauma is infrequently encountered; however, it can have fatal consequences if managed inappropriately. This paper provides an overview of the relatively limited literature available on the subject and highlights current controversies and recent advances in the management of these injuries.

## 1. Introduction

Compared to the adult population, blunt laryngotracheal trauma is rarely encountered in the paediatric age group [1]. This is believed to be due to behavioural factors as well as anatomic differences between these two groups [2]. The management of this clinical entity poses a particular challenge to the managing physician due to the fact that these patients often have very little signs and symptoms on initial presentation as well as the significant risk of concomitant injury to other vital structures in close proximity to the larynx [3]. Therefore, a high index of suspicion and a low threshold for investigation and intervention are required if the significant morbidity and potentially fatal consequences of these injuries are to be avoided. The aim of this paper is to provide an overview of the relatively limited literature available on the subject as well as to highlight recent advances and current controversies.

## 2. Aetiology, Incidence, and Anatomical Considerations

Laryngeal trauma is encountered in less than 1% of all patients with significant blunt trauma [4], and the incidence of laryngotracheal injuries in the adult population has been reported to account for between 1 and 12 in 30000 acute presentations to the emergency department [5, 6]. Whilst the specific incidence in the paediatric age group is yet to be reported, it is universally accepted that it is significantly lower than the rates quoted for the adult population [7]. Furthermore, it has been suggested that the severity of these injuries in children is often less severe than those sustained by their adult counterparts [2]. 

The lower incidence in the paediatric subset of patients has been attributed to a combination of both behavioural and anatomical factors. Perhaps the two most prominent behavioural factors are the fact that children are less frequently involved in road traffic accidents, in some part due to societal regulations, and they are also less likely to become involved in violent interpersonal conflicts [1, 4]. The paediatric larynx also benefits from certain anatomical advantages when compared to the adult larynx. The superior position of the paediatric larynx in the cervical region as well as a relatively short neck allows the mandibular arch to shield the larynx to some extent. Furthermore, the greater pliability of the cartilage decreases the likelihood of fractures, which in turn decreases the severity of injury [5, 8]. These factors are, however, offset to some degree by the narrow lumen of the paediatric airway as well as the increased risk of substantial oedema and haematoma formation due to the loose adherence of the submucosal tissue to the perichondrium of the laryngeal structure [2, 8]. 

The aetiology of blunt laryngeal trauma in paediatric patients is age dependent to some extent. Prepubertal patients typically sustain injuries inside the home environment, for instance, striking furniture during falls or handlebar accidents during cycling [8]. During adolescence, patients typically become more mobile and are exposed to risk factors more commonly encountered in the adult population. In this age group, motor vehicle accidents, sports injuries, and clothesline injuries, which typically occur during motorized vehicle accidents such as minibikes, snowmobiles, and jetskis, are commonly encountered and can lead to laryngotracheal separation [9].

## 3. Initial Management and Evaluation

The presenting symptoms of blunt laryngeal trauma are a varied spectrum which ranges from mild dysphonia to stridor and respiratory distress. Other symptoms may include dysphagia, haemoptysis, anterior neck pain, dyspnoea, and odynophagia [5, 8–10]. It is, however, of the utmost importance to realise that whilst patients with significant blunt laryngeal trauma are often relatively asymptomatic on initial presentation, they can rapidly develop debilitating stridor and subsequent respiratory compromise. A high index of suspicion and a low threshold for investigation are, therefore, required if the potentially fatal consequences of these injuries are to be avoided. Furthermore, it has been reported that early recognition and treatment of these injuries improves the long-term prognosis [3, 7]. On physical examination, particular attention should be paid to any of the following signs: anterior neck ecchymoses, loss of laryngeal landmarks, oedema, surgical emphysema, and palpable cartilage fractures [5, 10].

The initial management of these patients with laryngeal injuries depends on the stability of the patient's airway. Those patients whom are deemed to have an unstable airway on presentation should have a definitive airway placed. At present, there remains some controversy whether this is best achieved via endothracheal tube (ETT) placement [8, 11] or tracheotomy [1, 7, 10]. Proponents of surgical tracheotomy argue that endotracheal intubation can exacerbate mucosal lacerations, further disrupt already displaced structures and potentially lead to laryngotracheal separation or creation of a false passage. The most significant consideration, however, would be that failed endotracheal intubation will require an emergency tracheotomy, with potentially disastrous consequences [1, 10]. It has, however, been suggested that under certain circumstances ETT can be safely performed; this requires an experienced physician, excellent visualization of the airway, and a small ETT [1, 11]. Following a review of their case series Gold et al. recommend the use of a rigid bronchoscope to secure the airway under direct visualization following induction via inhalational anaesthetic agents in the operating room. Once the airway is secured, a surgical tracheotomy can then be performed over the bronchoscope [1]. Patients with a stable airway should be evaluated with flexible laryngoscopy in the first instance after which, microlaryngoscopy and bronchoscopy can be performed if indicated [3]. 

The radiographic assessment of these patients is guided, in part, by the risk of concomitant injury to vital structures in close proximity to the larynx, in particular the oesophagus and cervical spine. Investigations should include cervical spine X-rays, lateral soft tissue neck films, and potentially an oesophagogram with water-soluble contrast [11]. Up to 50% of patients with significant laryngeal trauma will have an associated cervical spine fracture [6], highlighting the importance of maintaining in-line stabilization of the cervical spine until it has been cleared. Whilst these patients infrequently develop a pneumothorax, a chest X-ray should be performed to out rule this and to determine whether subcutaneous emphysema or a pneumomediastinum is present, both of which are considered sensitive signs of laryngeal injury with an air leak [8].

Computed tomography (CT) provides the most valuable information regarding cartilage fractures of the larynx [8]. Whether or not it is indicated in all cases of blunt laryngeal trauma remains a relatively contentious issue. Following their review of 23 patients with blunt laryngeal trauma, Gold et al. recommend the use of CT scanning only in cases where the results may determine the management course [1]. The weight of evidence would, however, support the use of CT scanning in the evaluation of paediatric patients with blunt laryngeal trauma [6, 9–11]. The exception to this would be cases requiring an emergent tracheotomy and open exploration of the larynx [1, 7, 12]. 

## 4. Classification and Management

Laryngotracheal injuries are classified into 5 distinct groups based on the severity of the injury (Table 1) [2]. This ranges from minor endolaryngeal haematoma formation or laceration without detectable fractures in group 1 to complete laryngotracheal separation observed in group 5 [2]. Traditional management of patients in groups 1 and 2 consists of conservative measures including voice rest, humidification, prophylactic antibiotics, proton pump inhibitor therapy, and steroids [3]. Surgical intervention is reserved for patients falling into groups from 3 to 5. Recently, the endoscopic management of blunt laryngeal trauma has come to the fore, particularly in cases limited to substantial mucosal trauma. These techniques reduce both the morbidity associated with open surgery as well as the postoperative recovery time. Furthermore, it has been suggested that it can potentially avoid tracheotomy placement, in particular if the patient is kept intubated for 1 to 4 days postoperatively [9]. Open surgical exploration including laryngofissure with or without concomitant stent placement still has a role to play in cases with comminuted laryngeal cartilaginous fractures, injuries requiring visualization of the paraglottic space, extensive mucosal or vocal cord avulsion, and injuries that are not optimally visualized or repaired with endoscopic techniques [9]. Stenting is indicated in these cases if the anterior commissure is involved or disrupted, comminuted fractures and massive mucosal injuries [5]. If surgical exploration is to be undertaken, historical teaching considered it prudent to wait for 3–5 days [12]. Recent evidence would, however, suggest that earlier intervention improves the long-term outcome [3, 8, 9]. The long-term sequelae of paediatric blunt laryngeal trauma include persistent dysphonia, subglottic and rarely supraglottic stenosis, tracheotomy dependence, and the development of a tracheooesophageal fistula [2, 3, 9, 13]. 

## 5. Conclusion

Blunt laryngeal trauma is infrequently encountered in the paediatric population, something that is reflected by the limited literature available on the subject. These patients are often relatively asymptomatic on initial presentation; however, they can rapidly develop respiratory embarrassment, and, therefore, a high index of suspicion is required in order to manage these cases successfully. Whilst there remains some controversy on the topic, patients with an unstable airway require definitive management either via endotracheal intubation or tracheotomy. Recent advances in the management of these injuries include the increased use of endoscopic techniques, which in turn avoid the morbidity and potential complications associated with open exploration.

## Figures and Tables

**Table 1:** 

Group	Description
Group 1	Minor endolaryngeal haematoma or laceration without detectable fracture.
Group 2	Oedema, haematoma, and minor mucosal disruption without exposed cartilage.
Group 3	Massive oedema, mucosal tears, exposed cartilage, vocal cord immobility, and displaced fractures.
Group 4	Group 3 with more than 2 fracture lines or massive trauma to laryngeal mucosa.
Group 5	Laryngotracheal separation.
